# Entrustable professional activities as a framework for longitudinal geriatric oral health curriculum reform in undergraduate dental education

**DOI:** 10.3389/fdmed.2026.1847717

**Published:** 2026-08-31

**Authors:** Kiran Kumar Ganji, Shivasakthy Manivasakan, Mohammad Khursheed Alam, Mohammed Katib Alruwaili, Meshal Aber Alonazi, Hmoud Ali Algarni

**Affiliations:** 1 Department of Preventive Dentistry, College of Dentistry, Jouf University, Sakaka, Saudi Arabia; 2 Institute of Health Professions Education, Sri Balaji Vidyapeeth, Puducherry, India; 3 Department of Restorative Dental Sciences, College of Dentistry, Jouf University, Sakaka, Saudi Arabia

**Keywords:** curriculum reform, entrustable professional activities (EPA), geriatric dentistry, gerodontology, interprofessional education, undergraduate dental education

## Abstract

Population ageing is increasing the complexity of oral health care and creating new expectations for undergraduate dental education. Elder adults increasingly present with multimorbidity, frailty, polypharmacy, functional dependency, cognitive decline, and social vulnerability, all of which require dental graduates to move beyond procedural competence toward integrated, person-centred, and collaborative care. However, the literature continues to show substantial variability and underdevelopment in undergraduate gerodontology teaching, with inconsistent curricular structure, limited standardization, and insufficient attention to authentic clinical performance. Recent work in dental education also suggests that Entrustable Professional Activities (EPAs) may provide a practical bridge between competency frameworks and workplace-relevant assessment, yet their use in geriatric oral health education remains limited. This article presents a curriculum innovation framework for longitudinal integration of geriatric oral health in undergraduate dental education, informed by competency-based education, contextualized EPAs, constructive alignment, spiral curriculum design, interprofessional education, and programmatic assessment. On this basis, six context-sensitive geriatric oral health EPAs are proposed, together with a longitudinal curriculum model, suggested pedagogical approaches, and an assessment framework. Rather than being restricted to a single institutional curriculum, the model is intended as a flexible reform framework that can be adapted across diverse undergraduate BDS programs. EPA-informed curricular reform offers a theory-informed and practically relevant approach for making geriatric oral health teaching more explicit, longitudinal, interprofessional, and assessable in undergraduate dental education.

## 1 Introduction

Population ageing is fundamentally changing the nature of oral health needs and the competencies expected of newly qualified dentists from universities. The World Health Organization's current global agenda on oral health stresses that oral health is an integral part of universal health coverage, prevention of noncommunicable diseases, health systems strengthening and life-course health and is essential for healthy ageing, functional ability, nutrition, communication, social participation and well-being in later years (1, 2). At the same time, elder adults are maintaining more teeth and living longer, increasingly with medically and socially complex conditions, which influence their oral healthcare needs in the context of multimorbidity, frailty, polypharmacy, cognitive impairment, dependence on caregivers, access difficulties and social disadvantage (1, 3). In the context of gerodontology, these challenges necessitate that dentists possess not only clinical and technical proficiency but also skills in clinical reasoning, risk assessment, communication, ethics, prevention-oriented practice, priority setting and teamwork with health professionals. Unfortunately, despite these changing population needs, gerodontology education isn't consistently represented in undergraduate dental curricula. In a scoping review of dental school curricula, Nilsson et al. (4) reported considerable variation in the coverage of gerodontology content, the ways in which it was taught and the methods used for assessment. These authors concluded that clearer educational guidelines are needed to prepare dental graduates for the oral health needs of frail and care-dependent elder adults.

Similarly, contemporary curriculum literature argues that undergraduate education regarding elder adult oral health is often limited and requires revision that's more evidence-based, student-centred and integrated (5, 6). One reason for this disparity may be that conventional curricula often stop at competency statements. While competencies outline the characteristics that graduates are expected to possess, they frequently fall short of articulating how trainees should behave in true practice environments or how readiness for practice should be assessed when dealing with complex subject areas such as gerodontology. In response, Entrustable Professional Activities (EPAs) provide a promising curriculum solution. Dental educators are discussing the utilization of EPAs to transition broader competency frameworks into practical components of professional practice that can be taught, observed, overseen and sequentially entrusted (7). The applicability of EPAs to elder adult oral health is particularly germane, as caring for this demographic hinges not only on the trainee's knowledge base but also on its effective implementation within the real clinical, social and interprofessional context.

This article presents a theory-informed educational activity innovation framework for longitudinal integration of geriatric oral health in undergraduate dental education using contextualized EPAs.

## 2 Pedagogical frameworks, pedagogical principles, and competencies underlying the educational activity

This framework is grounded in an integration of competencies, EPAs, constructivist alignment, the spiral curriculum model, the inclusion of IPE and an outcome-based, programmatic evaluation approach (7, 8). EPAs operationalize geriatric oral health competencies in a way that makes the abilities testable, teachable and observable, though this practice remains underdeveloped in dental education (7). To address this, we propose a spiral curriculum structure with repetition and increasing authentic complexity and responsibility (5, 6) as part of a comprehensive, student-centred curricular reform of the elder adult dental curriculum. This also incorporates an interprofessional approach, because these patients often have multiple medical issues and thus are frequently managed in an interprofessional health care setting, where management failure has been associated with poor communication across health professions and poor coordination between them (3, 9). The plan is further guided by a programmatic assessment system which supports repeated assessment of learner ability over the span of the curriculum with different methods and the use of explicit conceptual models for curriculum development in a defensible, theory-based manner (10, 11). Together, these components provide complementary functions within the proposed educational design. Competency-based education defines the capabilities expected of graduating dental students, while the Age-Friendly Health Systems 4Ms establish the clinical and interprofessional dimensions of elder -adult care. The proposed EPAs translate these expectations into observable units of professional work; constructive alignment connects the EPAs with learning activities and assessment; spiral curriculum design supports repeated exposure with increasing complexity and responsibility; and programmatic assessment integrates evidence from multiple observations to inform developmental entrustment decisions. Their combined application therefore provides a coherent pedagogical rationale for organizing geriatric oral health education longitudinally rather than as isolated curricular content. what a newly qualified general dentist needs to be proficient in for geriatric patients

In geriatric oral health, the same clinical responsibility may mean different things for an independent, frail, or cognitively impaired elder adult, or one who's medically unstable, lives in an institution, or is homebound. Geriatric oral health must, therefore, be moved beyond general outcome competencies and instead specify specific skills that learners should develop, the specific settings in which learners will use them and the degree to which learners require supervision when using them. When these principles are applied to an undergraduate dental school program, the innovation hopes to build competencies in all aspects of the oral healthcare needs of elder adults; including assessment, treatment planning, interpersonal communication skills, oral health prevention, ethical and professional judgments, the detection of problems affecting oral health through medications, the relationship between systemic disease and oral health and the coordination of care across disciplines.

### 2.1 Framework-development approach and comparative curriculum scan

The development of the proposed curriculum framework was informed by contemporary evidence on Entrustable Professional Activities in dental education, gerodontology education, competency-based education, constructive alignment, spiral curriculum design, interprofessional education, programmatic assessment, and age-friendly healthcare. The educator-related dimensions of curriculum design, teaching, supervision, mentoring, assessment, and educational development were additionally informed by the scoping review of Abu Bakar et al., which synthesized elements of Entrustable Professional Activities for dental educators (12). Because that review addressed educator roles rather than student activities in geriatric oral healthcare, its findings were used to inform the educational and faculty-development components of the present framework and were combined with literature specific to elder -adult oral health, the Age-Friendly Health Systems 4Ms, and undergraduate dental education.To contextualize the proposed framework, a structured descriptive scan of publicly available curriculum information was conducted on 24th March 2026. Institutions occupying the top 10 positions in the QS World University Rankings by Subject 2026 (13).

Official institutional websites were examined for programme specifications, curriculum maps, course and module descriptions, syllabi, competency statements, clinical-rotation information, and descriptions of educational initiatives. Search concepts included “geriatric dentistry,” “gerodontology,” “elder adults,” “ageing,” “special care dentistry,” “medically compromised patients,” “comprehensive care,” “interprofessional education,” “community care,” “outreach,” and “domiciliary care.” Information was extracted on the presence and terminology of geriatric oral health content, curricular location, longitudinal integration, clinical exposure, interprofessional learning, community or home-based experience, and identifiable assessment approaches. Of the 10 institutions reviewed, four had clearly identifiable explicit geriatric courses, longitudinal initiatives, or structured clinical experiences; three demonstrated geriatric or ageing-related content embedded within broader comprehensive, special-care, or interprofessional curricula; two had identifiable gerodontology departments or specialist clinical infrastructure but insufficient information to establish the precise undergraduate curricular placement; and one provided insufficient public detail for confident classification. These findings demonstrate marked variation in the terminology, visibility, organization, clinical exposure, and assessment of geriatric oral health education across the selected programmes. Findings were summarized descriptively in Supplementary Table S1. When curricular information was unavailable or insufficiently detailed, the programme was categorized as “not identifiable from publicly available information,” and no assumption was made that geriatric oral health education was absent. The scan identified marked variability in the organization and visibility of geriatric oral health education. Four institutions had publicly identifiable explicit geriatric courses, rotations, or structured clinical experiences; three appeared to embed elder -adult oral health within broader special-care, comprehensive-care, or interprofessional curricula; one had specialist geriatric clinical infrastructure without sufficient information to determine its undergraduate curricular placement; and three could not be reliably classified from the available public information. The scan was descriptive and was intended to contextualize curriculum variation rather than rank programme quality or comprehensiveness.

## 3 Learning environment, learning objectives, and pedagogical format

### 3.1 Learning environment

The proposed educational activity is intended for undergraduate dental programs in which geriatric oral health is currently fragmented, embedded, or insufficiently developed across the curriculum. Oral health and ageing research makes it evident that care for elder adults cannot be effectively dealt with in a single lecture session, one module, or an optional elective course. Oral health in later life impacts on nutrition, swallowing, systemic disease, quality of life, medication impact, frailty trajectories and social support networks (2, 3). Such complexity cannot be dealt with by curricula based on disciplines without an adequate focus on patient-centred care. A longitudinal approach is required for a number of reasons. Firstly, ageing-related oral health issues involve many learning domains, including biomedicine, behavioural science, ethics, prevention, restoration, communication and teamwork. Secondly, students must be repeatedly exposed to these topics at increasingly complex levels as they progress from basic science to clinical settings. Thirdly, elder adult oral health is inherently contextual and thus must be rooted in realistic cases rather than unrelated facts. This is supported by Tabrizi and Lee (5), who suggested that links should be more deliberately formed between dentistry and gerontology to account for population ageing. Naka et al. (6) suggested the need for student-centred and integrated approaches to teaching dentistry to elder adults. Taken together, this research suggests that curricula should aim to restructure learning towards capabilities based on meaning, development and practice rather than simply adding content.

Rather than be the remit of just one course or institution's curriculum, the EPA-inspired revision of geriatric oral healthcare can be adopted as a linear framework applicable to many undergraduate dental programs worldwide, with teaching structured across three phases. This proposed model is intended for use in undergraduate dental education. It has potential applicability across the basic sciences curriculum, preclinical laboratory courses, preclinical clinical activities and comprehensive care experiences. The identified target audience is undergraduate dental students and the intended implementers include faculty members within the domains of oral medicine, restorative dentistry, community dentistry, ethics, behavioural sciences and clinical education, as well as interdisciplinary collaborators where they exist.

### 3.2 Learning objectives

By the end of the proposed curricular sequence, learners should be able to:

#### 3.2.1 Knowledge and understanding

- Recognize the influence of medical, functional, and social factors on the oral health needs of elder adults.
- Identify oral-systemic and medication-related risks relevant to the care of elder adults.

#### 3.2.2 Skills

- Assess the oral health needs of elder adults in relation to their medical, functional, and social context.
- Develop person-centred care plans for elder adults with frailty, multimorbidity, or dependency.
- Communicate effectively with elder adults, caregivers, and other health professionals.
- Adapt preventive and basic therapeutic care to the needs of vulnerable elder adults.

#### 3.2.3 Values

- Demonstrate ethical judgment in the management of elder adults.
- Demonstrate interprofessional collaboration in the management of elder adults.

### 3.3 Pedagogical format

**Figure 1:**
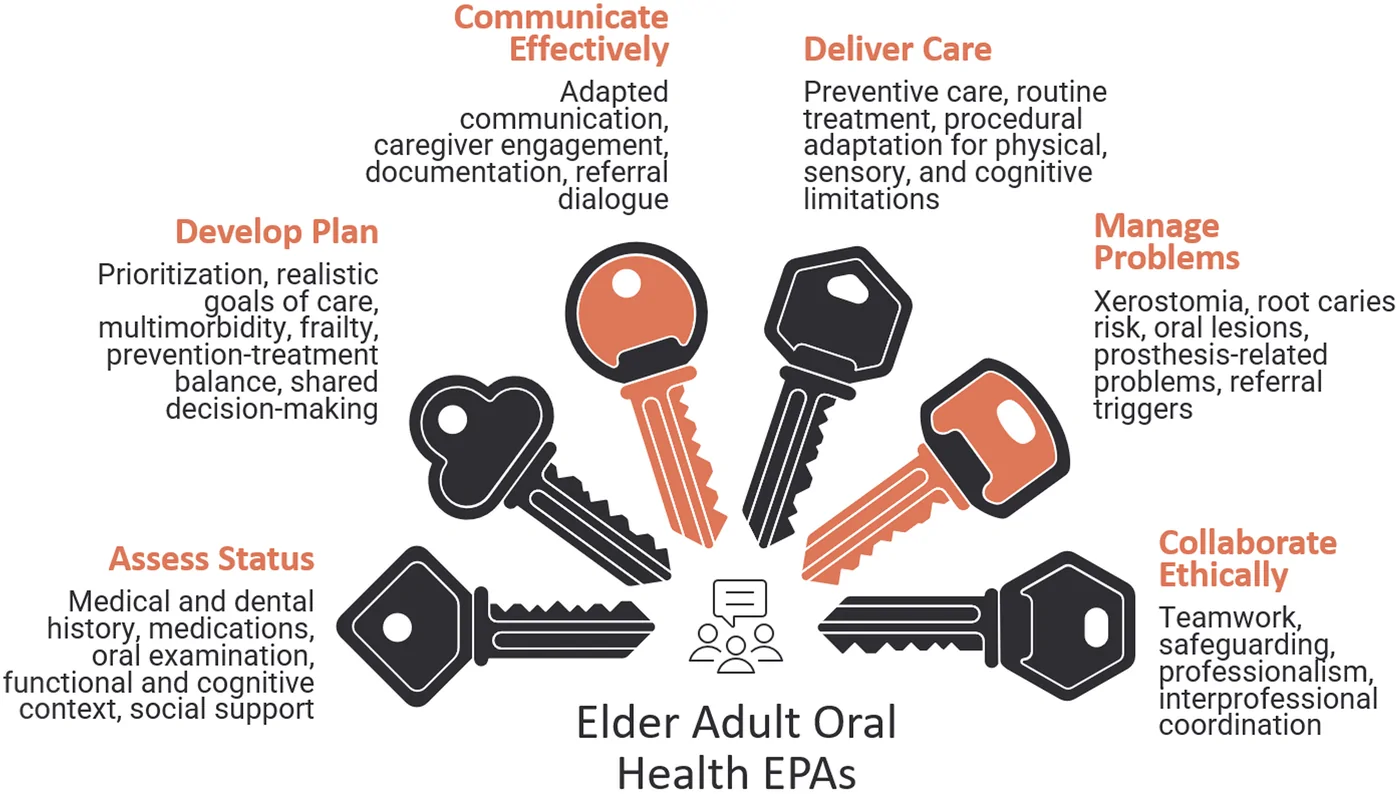
Proposed geriatric oral health EPAs and core descriptors.

One practical method to institutionalize such change involves developing a list of geriatric oral health EPAs, which can serve as longitudinal curriculum markers throughout undergraduate dental programs. Instead of replacing competencies, EPAs frame them as activities relevant to authentic professional duties and serve to delineate what a newly qualified general dentist needs to be proficient in for geriatric patients, with the understanding that undergraduate performance is supervised and educational (Figure 1).

These EPAs are intentionally contextualized. They reflect the multidimensional nature of elder adult oral health care described in recent literature, including polypharmacy, dependency, caregiver involvement, prevention, and integration with broader health and social care systems (1, 3, 14).

#### 3.3.1 Interprofessional framework and healthcare-delivery pathway

The Age-Friendly Health Systems 4Ms framework: What Matters, Medication, Mentation, and Mobility provides the evidence-based clinical and interprofessional care-delivery foundation for the proposed curriculum innovation. The 4Ms are intended to be addressed together to align care with the priorities of elder adults, optimize medication use, recognize and manage mentation-related concerns, and support safe mobility and functional ability across healthcare settings. Their application to geriatric oral health education is supported by emerging dental education evidence demonstrating the relevance of the 4Ms for preparing dental learners to care for medically and functionally complex elder adults. Within the proposed model, the 4Ms define the essential dimensions of age-friendly care, whereas the geriatric oral health EPAs translate these dimensions into authentic, observable, and assessable professional activities that students can learn, practise, and progressively perform with appropriate supervision (5, 15, 16).

Within this integrated framework, **What Matters** requires students to identify the elder adult's goals, preferences, concerns, and acceptable treatment burden and to incorporate these considerations into shared oral healthcare planning. **Medication** requires systematic medication review and recognition of medication-related oral effects, such as xerostomia, bleeding risk, altered healing, and drug interactions, with consultation or referral to physicians and pharmacists where necessary. **Mentation** requires students to recognize cognitive impairment, delirium, depression, communication difficulties, and the possible need for caregiver involvement or modified consent procedures. **Mobility** requires consideration of physical function, falls risk, transportation, accessibility, treatment positioning, ability to perform oral hygiene, and the potential need for adapted or domiciliary care.

The proposed healthcare-delivery pathway begins with a structured dental and 4Ms-informed assessment. Identified needs are then translated into a person-centred oral healthcare plan, followed by communication, referral, or joint decision-making with relevant professionals. Depending on the patient's needs and the local healthcare context, the interprofessional team may include physicians, nurses, pharmacists, physiotherapists, occupational therapists, dietitians, social workers, speech and language therapists, caregivers, and community health personnel. Dental students would participate at a level appropriate to their stage of training and under defined faculty supervision.

The framework may be delivered through interprofessional case conferences, case-based learning, simulated consultations, structured referral and handover exercises, supervised comprehensive-care clinics, community outreach, and placements in facilities serving elder adults. Follow-up and feedback from patients, caregivers, dental supervisors, and collaborating professionals would provide evidence of student progression. This pathway provides a practical mechanism for integrating oral health into the broader care of elder adults while allowing implementation to be adapted to local staffing, resources, referral systems, and clinical opportunities.

**Figure 2:**
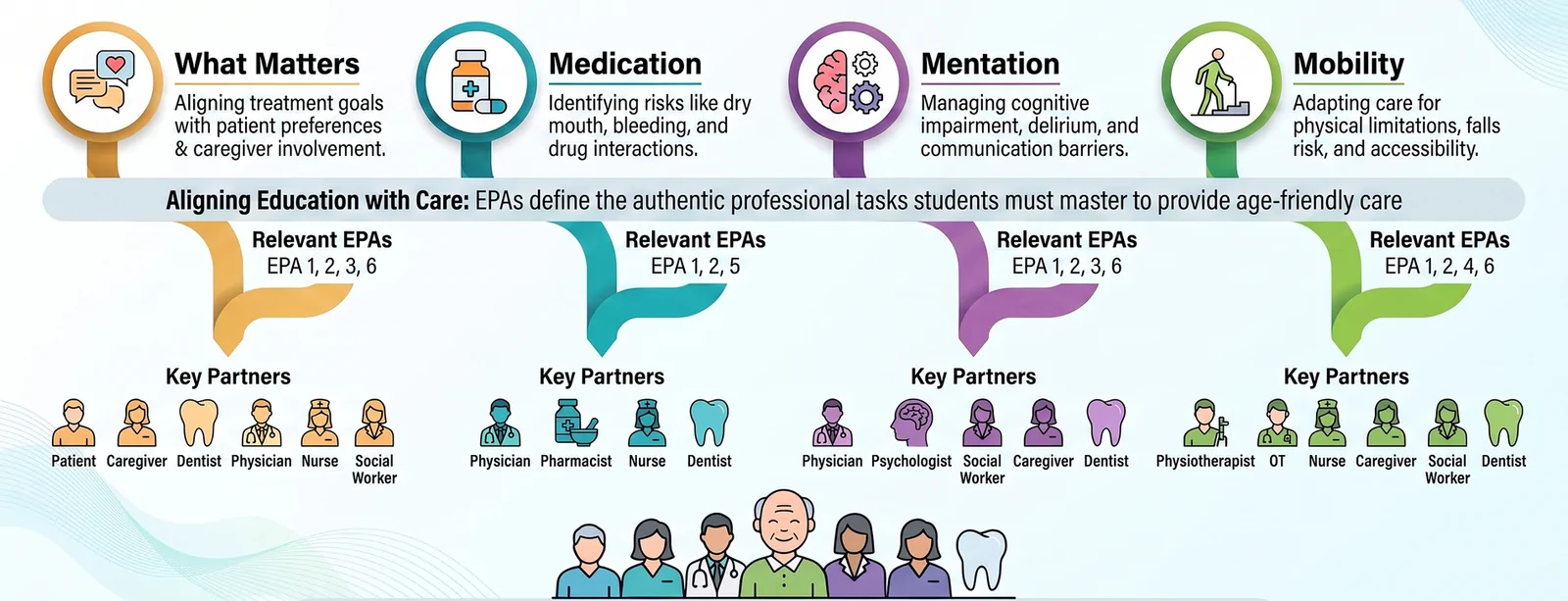
Integrated EPA–4Ms framework for longitudinal geriatric oral health curriculum implementation.

The 4Ms would therefore be embedded across the three longitudinal phases rather than delivered as an isolated module, progressing from foundational recognition and case-based application to supervised interprofessional care planning and workplace-based clinical performance. In the first phase, students will be exposed to basic aspects of ageing, common oral diseases in the elder population, oral-systemic relationships, effective communication skills, relevant professional ethics and psychosocial factors. In the second phase, students will be introduced to basic adapted assessment of elder people, prevention strategies, basic treatment planning and patient referral in a simulated setting and supervised real patient settings. In the third phase, these newly acquired skills will be enhanced by allowing students to perform comprehensively, managing patients with complex needs and developing clinical judgement in a real patient setting (Figure 2). This tiered approach combines aspects of a spiral curriculum with the need to contextualize geriatric care at different stages of training (5, 6). This model is meant to be adaptable rather than prescriptive. Different schools may place these EPAs in different years or courses depending on local sequencing, staffing, and scope of practice, but the longitudinal logic remains applicable across varied undergraduate dental program structures.

If education concerning elder adult oral health is to become an authentic practice, pedagogical techniques must advance beyond lecture-format and begin fostering the ability of students to use context, form judgments, articulate views and integrate different bodies of information. Teaching and learning strategies that prioritize activity and are context-based and clinic-centric, in keeping with recent literature regarding gerodontic education (2, 6), should continue to be integrated. Lectures and seminars should still be used to cover fundamental topics of ageing, frailty, links between oral and systemic health, the effects of medication and the complexities of ethical decision-making. However, students can be brought closer to the reality of providing care for elder adults through the use of case-based learning and by exposing them to complex case scenarios involving elder adults who are multimorbid, dependent on others, are on multiple medications, experience xerostomia, have a higher risk of root caries and require treatment to be prioritized. Simulation and objective structured clinical examinations (OSCEs) are important for teaching communication skills with elder adults, while service learning and outreach can give students deeper insight into accessibility issues, preventative health strategies and care networks (5, 9, 14).

### 3.4 A longitudinal model for integration across undergraduate dental education

Rather than be the remit of just one course or institution's curriculum, the EPA-inspired revision of geriatric oral healthcare can be adopted as a linear framework applicable to many undergraduate dental programs worldwide, with teaching structured across three phases. In the first phase, students will be exposed to basic aspects of ageing, common oral diseases in the elder population, oral-systemic relationships, effective communication skills, relevant professional ethics and psychosocial factors. In the second phase, students will be introduced to basic adapted assessment of elder people, prevention strategies, basic treatment planning and patient referral in a simulated setting and supervised real patient settings. In the third phase, these newly acquired skills will be enhanced by allowing students to perform comprehensively, managing patients with complex needs and developing clinical judgement in a real patient setting (Table 1). This tiered approach combines aspects of a spiral curriculum with the need to contextualize geriatric care at different stages of training (5, 6). This model is meant to be adaptable rather than prescriptive. Different schools may place these EPAs in different years or courses depending on local sequencing, staffing, and scope of practice, but the longitudinal logic remains applicable across varied undergraduate dental program structures.

**Table 1:** Proposed longitudinal mapping of geriatric oral health EPAs across undergraduate dental education.

EPA	Introduced	Practiced	Consolidated
EPA 1: Assess the elder adult	Foundational biomedical sciences, behavioural sciences, community dentistry	Introduction to clinical practice, case-based teaching, supervised clinical encounters	Comprehensive care/total patient care
EPA 2: Person-centred care planning	Dental sciences and treatment-planning foundations	Clinical case discussions, oral medicine/oral surgery teaching, problem-solving courses	Comprehensive care/total patient care
EPA 3: Communication with patients/caregivers/professionals	Communication skills, behavioural sciences, community dentistry	Ethics, simulated communication, referral and documentation activities	Comprehensive care/total patient care
EPA 4: Deliver preventive/basic care with modification	Preclinical skills and prevention teaching	Skills laboratory, supervised clinic, prevention-focused case management	Senior clinical practice/comprehensive care
EPA 5: Recognize oral-systemic/medication-related problems	Pathology, medicine for dentistry, emergency teaching	Oral medicine, medically compromised patient cases, referral exercises	Senior clinical reasoning/comprehensive care
EPA 6: Interprofessional and ethical collaboration	Community and professionalism teaching	Ethics, interprofessional case discussions, outreach/community experiences	Senior clinical practice/practice management/comprehensive care

### 3.5 Proposed implementation strategy, timeline, and resource considerations

Implementation of the proposed curriculum innovation should proceed through a phased and context-sensitive process rather than immediate programme-wide adoption. During an initial two-month planning phase, programme leaders and a multidisciplinary curriculum working group would assess existing geriatric oral health teaching, clinical exposure, interprofessional opportunities, referral pathways, faculty capacity, and institutional readiness, followed by a two-month curriculum-mapping and co-design phase to align existing learning activities and assessments with the six geriatric oral health EPAs and the 4Ms, identify gaps and duplication, and agree stage-appropriate learning outcomes, supervision expectations, and referral pathways. These educator functions, including curriculum development, teaching, clinical supervision, mentoring, feedback, and assessment, are consistent with the EPA domains for dental educators identified by Abu Bakar et al. (12). Faculty and assessor development would then be undertaken over approximately two months, addressing application of the 4Ms, facilitation of interprofessional learning, direct observation of EPA performance, entrustment decision-making, feedback, and use of assessment rubrics. Initial implementation could subsequently be piloted over six months within one cohort, course, clinical setting, or selected group of EPAs, using existing teaching sessions, case-based learning, simulation, routine clinical encounters, referral exercises, and portfolio activities wherever feasible. Evaluation and refinement could be completed over the following three months using learner outcomes, entrustment data, 4Ms completion, implementation fidelity, faculty and student feedback, and feasibility findings, followed by a further three-month scale-up phase across relevant years and clinical settings, with ongoing monitoring and curriculum quality improvement. The complete planning-to-scale-up period is therefore estimated at approximately 18 months, although the duration should be adapted to local approval processes, programme structure, faculty capacity, and access to elder -adult patients and interprofessional partners. The framework is designed to minimize additional expenditure by using existing courses, clinical facilities, faculty expertise, case materials, routine patient encounters, and established referral systems; essential resource requirements would primarily include protected faculty time, administrative coordination, curriculum mapping, faculty and assessor development, teaching and assessment materials, data management, and programme evaluation, while additional costs may arise from standardized patients, advanced simulation, digital portfolio platforms, external interprofessional facilitators, community transport, domiciliary visits, or outreach placements. Because salaries, infrastructure, patient-care systems, and educational costs vary substantially across institutions and countries, universal monetary estimates would be inappropriate; each institution should therefore undertake a local budget-impact assessment during planning, monitor actual resource use during the pilot, and use these findings to determine the feasibility and sustainability of wider implementation.

## 4 Results to date and assessment

To date, this educational activity innovation has yielded a six-EPA framework for geriatric oral health, a proposed three-phase longitudinal model for undergraduate integration, and aligned pedagogical and assessment strategies. These outputs should be interpreted as curriculum design products rather than implementation outcomes. EPAs also offer a better measure of aptitude than only considering the extent of content coverage. Geriatric oral health requires assessment of more than simply knowledge retention as demonstrated by written exams. Competence is much more than theoretical understanding, it also includes an element of judgment, sensitivity to context, good communication skills and a high degree of professionalism and adapted care. Programmatic assessment is therefore a suitable approach to the assessment of geriatric oral health. The emphasis should be placed on a range of assessment types across an entire program, rather than on high-stakes assessments, so that learners can be assessed on a number of different occasions based on a series of different observations and sources of evidence (10). During the early stages of a curriculum, knowledge and case interpretation could be prioritized, with an emphasis on structured communication and care planning tasks for the intermediate years and observations within the workplace supplemented by portfolio evidence for final-year students (Table 2).

**Table 2:** Suggested pedagogical & assessment strategies per EPA.

EPA	Pedagogical formats	Assessment strategies		
		Early stage	Mid stage	Senior stage
EPA 1	Structured history taking, medication review, oral-systemic seminars, frailty case discussions	MCQs, short cases, structured history exercises	OSCE medication review and case analysis	Direct observation and case-based oral examination
EPA 2	Treatment-planning workshops, shared decision-making exercises, case-based prioritization	Written treatment-planning tasks	Structured case presentations	Comprehensive care planning assessment
EPA 3	Role-play, caregiver communication OSCEs, referral writing, interprofessional case conferences	Role-play, communication presentations	Communication OSCEs	Direct observation, documentation and referral review
EPA 4	Skills-lab adaptations, preventive care planning, supervised clinical encounters	Skills-lab checklists	OSCE/OSPE adaptation tasks	Observed clinical care
EPA 5	Scenario-based learning, oral medicine seminars, medication-risk exercises, referral thresholds	Scenario-based MCQs	Case discussions and referral stations	Workplace-based assessment and case portfolio
EPA 6	Ethics seminars, team-based cases, community-based learning, collaborative care discussions	Reflective writing	Interprofessional case analysis	Portfolio and professionalism review

A simple undergraduate entrustment scale may also help make progression more explicit: (1) Observation only, (2) Performs with direct proactive supervision, (3) Performs with reactive supervision immediately available, (4) Performs with indirect supervision appropriate to undergraduate limits. Such a scale should be interpreted developmentally and not as a claim of independent specialist practice.

Because the proposed curriculum addresses several dimensions of performance, its outcomes should be evaluated through a multimethod assessment framework rather than a single instrument. The principal educational outcome would be students' progression in EPA performance and level of supervision, measured through repeated structured observation using an EPA-specific entrustment rubric. Knowledge, clinical reasoning, communication, interprofessional collaboration, and implementation of the 4Ms would be evaluated as complementary secondary outcomes. Evidence from different assessment methods, assessors, clinical contexts, and stages of training would be reviewed programmatically to determine learner progression and identify areas requiring additional supervision or educational support. The proposed outcome domains and measurement tools are summarized in Table 3.

**Table 3:** Proposed outcome domains and measurement tools for evaluating the curriculum framework.

Outcome domain	Proposed measurement tool	Suggested indicator
EPA performance and entrustment	EPA-specific direct-observation rubric using the four-level supervision scale	Change in EPA performance and supervision level across repeated observations
Knowledge and clinical reasoning	Pre- and post-intervention written assessment and structured case analysis	Change in knowledge and clinical-reasoning scores
Communication and person-centred care planning	OSCE checklist or analytic rubric	Performance in communication, shared decision-making, and care-planning stations
Interprofessional collaboration	Analytic rubric aligned with the IPEC domains of values and ethics, roles and responsibilities, communication, and teams and teamwork	Domain scores obtained during case conferences, referral exercises, simulations, or clinical encounters
Delivery of the 4Ms	Structured 4Ms assessment, documentation, and action checklist	Proportion of encounters in which What Matters, Medication, Mentation, and Mobility are assessed, documented, and acted upon
Longitudinal development	Reflective portfolio, case documentation, and multisource feedback	Evidence of progression across different stages and clinical contexts
Feasibility and acceptability	Structured learner and faculty questionnaire, supplemented by interviews or focus groups where appropriate	Acceptability, feasibility, perceived relevance, and implementation barriers
Implementation fidelity	Curriculum implementation checklist	Proportion of planned learning activities delivered, faculty participation, and student exposure to elder -adult care
Patient or caregiver experience	Brief experience or satisfaction questionnaire, where feasible	Perceptions of communication, involvement in decisions, coordination, and responsiveness of care

The outcomes of the proposed curriculum innovation would be organized and interpreted using a modified Kirkpatrick model (Table 4), supplemented by implementation measures to account for feasibility, fidelity, resources, and institutional context (17, 18).

**Table 4:** Proposed evaluation of the curriculum innovation using a modified Kirkpatrick model.

Evaluation level	Outcome focus	Proposed indicators	Measurement methods
Level 1: Reaction	Learner and faculty perceptions of the educational activity	Relevance, acceptability, perceived usefulness, satisfaction, feasibility, and confidence in caring for elder adults	Structured learner and faculty questionnaires, reflective feedback, and focus-group discussions
Level 2: Learning	Acquisition of knowledge, clinical reasoning, communication, and interprofessional competence	Changes in geriatric oral health knowledge, case interpretation, person-centred care planning, communication, and collaborative practice	Pre- and post-intervention written assessments, structured case analyses, OSCEs, and IPEC-aligned analytic rubrics
Level 3: Behaviour	Application of learning in simulated or authentic clinical practice	Performance of the proposed EPAs, progression in entrustment level, quality of referrals, documentation, communication, and coordinated care planning	Repeated direct observation, EPA-specific entrustment rubrics, workplace-based assessment, case-based discussion, portfolio evidence, and multisource feedback
Level 4: Results	Practice-related and patient-care process outcomes	Completion of 4Ms-informed assessment, appropriate interprofessional referrals, coordinated care plans, continuity of follow-up, and patient or caregiver experience	4Ms assessment-documentation-action checklist, referral audit, care-plan review, follow-up records, and brief patient or caregiver experience questionnaires
Supplementary implementation evaluation	Context, feasibility, fidelity, and sustainability	Faculty participation, learner exposure, adherence to planned activities, resource requirements, implementation barriers, and facilitators	Implementation checklist, activity logs, faculty and learner surveys, interviews, and focus groups

## 5 Discussion: practical implications, objectives and lessons learned

Many elder adults, particularly those who are frail, medically complex, or live in the community or at home, are dependent on intra-professional communication as well as communication among team members and with caregivers, support networks and the like. This was demonstrated well by Hoste et al. (9) when they noted that interprofessional communication for frail elder adults was often prevented by a siloed system, poorly defined professional roles and the failure of primary care stakehelder s to communicate. Undergraduate dental curricula must, therefore, prepare the learner to work in collaborative arrangements with colleagues in other disciplines and non-professional caregivers and support networks, and not just to provide a range of dental care interventions without this coordination. The EPA-informed approach to assessment is useful in this context because it places communication, referral, sharing of clinical decision-making and collaborative care planning as part of the learner's expected professional work. The integration of the proposed EPAs with the 4Ms framework provides an operational bridge between dental education and age-friendly healthcare delivery by connecting oral assessment and treatment planning with patient priorities, medication-related risks, cognitive status, functional ability, interprofessional referral, and coordinated follow-up. In this way, the learner is being taught to think of interprofessional learning not as an extra or an option, but as an integral part of a complete and competent education in geriatric oral health.

This EPA framework doesn't require every school to create a distinct course in geriatric dentistry, nor is it intended to prescribe a particular sequence of courses within a undergraduate dental program. It does provide a mechanism for making geriatric oral health more visible in undergraduate dental programs and for assessing and providing longitudinal education within any given curriculum format. This is important because, in many dental schools, teaching regarding the oral medicine of elder adults, the community dentistry aspects of providing care for elder adults, communication, ethics, restorative planning and a focus on comprehensive care already exists, but there's no coherent framework to link these components into a progressive preparation to care for elder adults. EPA-informed reform can provide this framework (Table 5).

**Table 5:** Integrated theoretical framework and curricular implications.

Framework element	Why it matters	Curricular implication
Competency-based education and EPAs	Translates broad outcomes into practice	Define geriatric EPAs as authentic units of work
Contextualization	Elder adult care varies by frailty, dependency, cognition, and setting	Frame EPAs around real geriatric contexts
Constructive alignment	Links outcomes, teaching, and assessment	Align learning activities and assessment to EPAs
Spiral curriculum	Revisits learning with increasing complexity	Stage geriatric learning across years
Interprofessional education	Reflects real elder adult care pathways	Include team-based cases and referral practices
Programmatic assessment	Supports developmental judgment over time	Use multiple observations and evidence sources

A key lesson from the literature and comparative curriculum scan is that geriatric oral health education is represented across leading dental institutions but varies substantially in terminology, curricular visibility, delivery setting, and degree of longitudinal integration. Publicly identifiable approaches ranged from standalone courses and structured geriatric clinical experiences to content embedded within special-care dentistry, comprehensive care, or interprofessional education. In several institutions, the available public information was insufficient to establish the precise curricular location or assessment of elder -adult oral health. These findings reinforce the need for a flexible framework that can make geriatric oral health expectations more explicit, developmental, and assessable without prescribing a uniform course structure. Communication, collaboration, and contextualized care should therefore be embedded across the curriculum rather than confined to isolated or advanced clinical experiences.

## 6 Acknowledgment of conceptual, methodological, environmental, and material constraints

This article presents a curriculum innovation framework rather than an implemented educational intervention. The comparative curriculum scan was limited to publicly available information from a purposive sample of highly ranked dental institutions. Public programme pages and course descriptions may be incomplete, outdated, unavailable in English, or less detailed than internal curriculum documents; therefore, failure to identify a curricular component should not be interpreted as evidence of its absence. The scan was descriptive, did not involve direct verification with institutional representatives, and was not intended to evaluate or rank the quality of individual programmes. Delphi validation, broad consensus development and pilots of the EPA proposals weren't yet completed. Thus, this EPA framework should be regarded as transferable knowledge in concept and curricula, not as an established benchmark. Future research should address three key components: the validation and refinement of the proposed EPAs via consensus methods involving experts, pilot testing of the proposed EPA framework at multiple undergraduate dentistry departments to explore feasibility, acceptability and adaptability and education research examining learner results, faculty attitudes and implementation issues among different curricular contexts. Implementation may also be influenced by faculty development needs, variation in access to elder -adult clinical experiences, and differences in institutional curricular structure and resources. These key components and processes will ultimately determine how effectively the proposed framework may support broader reforms in geriatric oral health education curricula.

## 7 Conclusion

By making elder adult oral health teaching more explicit, developmental, and practice-oriented, EPA-informed curriculum reform has the potential to strengthen the preparedness of future dental graduates for the complexities of caring for ageing populations.

## Data Availability

The original contributions presented in the study are included in the article/Supplementary Material, further inquiries can be directed to the corresponding author.

## References

[1] Chan AKY Chu CH Ogawa H Lai EH-H. Improving oral health of elder adults for healthy ageing. J Dent Sci. (2024) 19(1):1–7. 10.1016/j.jds.2023.10.018 38303786 PMC10829734

[2] Poudel P Paudel G Acharya R George A Borgnakke WS Rawal LB. Oral health and healthy ageing: a scoping review. BMC Geriatr. (2024) 24(1):33. 10.1186/s12877-023-04613-7 38191307 PMC10773108

[3] Wongiam N Praditpornsilpa K Vacharaksa A. Comprehensive geriatric assessment for oral care in elder adults: a focus group study. BMC Geriatr. (2025) 25(1):232. 10.1186/s12877-025-05854-4 40200164 PMC11977952

[4] Nilsson A Young L Glass B Lee A. Gerodontology in the dental school curriculum: a scoping review. Gerodontology. (2021) 38(4):325–37. 10.1111/ger.12555 33977554

[5] Tabrizi M Lee WC. Linking current dental education to gerontological education to meet the oral health needs of growing aging populations. Front Oral Health. (2023) 4:1232489. 10.3389/froh.2023.1232489 37876529 PMC10591445

[6] Naka O Chatzidou P Pezarou LC Anastassiadou V. Reforming dental curricula: a student-centred novel approach integrating prosthodontic care for elder adults. Oral. (2025) 5(4):73. 10.3390/oral5040073

[7] Ehlinger C Fernandez N Strub M. Entrustable professional activities in dental education: a scoping review. Br Dent J. (2023) 234(3):171–6. 10.1038/s41415-023-5503-8 36765231

[8] Mahzari M AlNahedh T Ahmed AA A Rumyyan A Shaban S Magzoub ME. Practical guide to undergraduate medical curriculum alignment and mapping. Adv Med Educ Pract. (2023) Volume 14:1001–12. 10.2147/AMEP.S424815 PMC10516198 37745032

[9] Hoste N Baele E De Vleeschauwer A Mertens F Poppe L Janssens B. Interprofessional collaboration on oral health for frail home-dwelling elder people: a focus group study on needs and barriers experienced by general practitioners and community pharmacists. BMC Prim Care. (2025) 26(1):253. 10.1186/s12875-025-02955-2 40813971 PMC12351776

[10] Jamieson J Palermo C Hay M Bacon R Lutze J Gibson S. An evaluation of programmatic assessment across health professions education using contribution analysis. Adv Health Sci Educ. (2026) 31:211–38. 10.1007/s10459-025-10444-5 PMC12929344 40465148

[11] Yin X Wang L Li Q Han J Zhou F Wang S. Theoretical models to guide undergraduate medical curriculum development: an integrative review. BMC Med Educ. (2025) 25(1):1043. 10.1186/s12909-025-07644-3 40652251 PMC12255110

[12] A Bakar N Roslan NS Haq MAU Yusoff MSB. Elements of entrustable professional activities for dental educators: a scoping review. BMC Med Educ. (2025) 25(1):1481. 10.1186/s12909-025-07888-z 41126113 PMC12541977

[13] (QS) QS. QS World University Rankings by Subject: Dentistry: QS Quacquarelli Symonds (2026). Available online at: https://www.topuniversities.com/university-subject-rankings/dentistry (Accessed March 24, 2026).

[14] Asanza DM Njoku A Baviskar S Evans MA Mouloudj K. Oral hygiene care of elder adults and caregiver education: a systematic review. Hygiene. (2025) 5(4):50. 10.3390/hygiene5040050

[15] Cacchione PZ. Age-friendly Health Systems: The 4Ms Framework. Los Angeles, CA: Sage Publications Sage CA (2020). p. 139–40. 10.1177/1054773820906667 32036695

[16] Institute for Healthcare Improvement. Age-friendly Health Systems: Guide to Using the 4M's in the Care of Elder Adults in Hospitals and Ambulatory Practices. Boston: John A Hartford Foundation and Institute for Healthcare Improvement (IHI) (2020).

[17] Allen LM Hay M Palermo C. Evaluation in health professions education—is measuring outcomes enough? Med Educ. (2022) 56(1):127–36. 10.1111/medu.14654 34463357

[18] Brashers V Phillips E Malpass J Owen J. Measuring the impact of interprofessional education (IPE) on collaborative practice and patient outcomes. In: Institute of Medicine, editor. Measuring the Impact of Interprofessional Education on Collaborative Practice and Patient Outcomes. Washington: National Academies Press (US) (2015). p. 67–133. 10.17226/21726 26803876

