# Supplementary material for: Entrustable professional activities as a framework for longitudinal geriatric oral health curriculum reform in undergraduate dental education
Source: Front Dent Med. 2026 Aug 31;7:1847717. doi: 10.3389/fdmed.2026.1847717 (PMC13572119; doi:10.3389/fdmed.2026.1847717)
Supplement: Supplementary file 1 [file Table1.docx]

**Supplementary Table S1**

| **Institution** | **Publicly identifiable geriatric oral health component** | **Curricular location and delivery** | **Clinical or interprofessional component** | **Publicly identifiable assessment** | **Classification** |
| --- | --- | --- | --- | --- | --- |
| **University of Michigan–Ann Arbor, USA** | The DDS curriculum includes a named course, **Special Care Dentistry–Geriatrics**, providing a lecture-based introduction to the biological, psychological, sociological, behavioural, medical, and oral health aspects of ageing. | Located in the third year and supported by special-care dentistry, medically compromised-patient education, hospital dentistry, and comprehensive-care experiences. | The hospital-dentistry rotation includes treatment planning for complex patients, interprofessional interaction, clinical observation, assistance, and direct patient care where appropriate. | The geriatric course is curriculum-based; related hospital rotations use faculty evaluations, documented patient encounters, self-assessment, and reflective assignments. | **Explicit geriatric component** ([University of Michigan Dentistry](https://dent.umich.edu/education/academic-programs/doctor-dental-surgery/curriculum?utm_source=chatgpt.com)) |
| **University of Hong Kong, Hong Kong SAR** | The public BDS overview describes an integrated, student-centred curriculum covering biomedical, behavioural, social, and clinical sciences, but no separately named geriatric dentistry course was identified in the publicly accessible programme information reviewed. | Geriatric oral health may be incorporated within integrated clinical learning, but its precise location and longitudinal sequence could not be established from the public information. | The curriculum emphasizes integration, interpersonal communication, clinical learning, and comprehensive professional competencies. | A final-year portfolio includes case presentations, reports, and reflections, although geriatric-specific assessment was not identifiable. | **Not identifiable from publicly available information** ([HKU Faculty of Dentistry](https://facdent.hku.hk/learning/undergraduate.php?utm_source=chatgpt.com)) |
| **Academic Centre for Dentistry Amsterdam, Netherlands** | From the 2025–2026 academic year, **Oral care for vulnerable older adults at home** became a formal component of the dental curriculum. | Master’s-level dental students undertake a structured placement involving home visits to vulnerable older adults unable to attend conventional dental practices. | Students work in pairs and provide preventive oral healthcare in the older adult’s home, connecting dental education with community-based and domiciliary care. | The official announcement confirms a formal placement, but detailed assessment criteria were not publicly described. | **Explicit geriatric clinical component** ([ACTA](https://acta.nl/nl/onderzoek/kwetsbare-ouderen-krijgen-hulp-van-acta-studenten?utm_source=chatgpt.com)) |
| **King’s College London, United Kingdom** | The BDS curriculum includes integrated care, adult oral health, public health, whole-patient care, professionalism, and clinical practice; however, no explicitly named geriatric dentistry or gerodontology module was identifiable from the public module listing reviewed. | Older-adult care may be incorporated within adult oral health, integrated care, whole-patient care, or clinical placements, but this could not be confirmed as a distinct curricular strand. | The programme emphasizes whole-patient care, teamwork, clinical placements, and consideration of patients’ total dental and medical needs. | Publicly described assessments include e-portfolios, written examinations, case presentations, structured clinical reasoning examinations, OSCEs, and continuing competence-based clinical assessment, but no geriatric-specific assessment was identified. | **Embedded or potentially embedded content; geriatric placement not publicly explicit** ([King's College London](https://www.kcl.ac.uk/study/undergraduate/courses/dentistry-bds/teaching-and-modules?utm_source=chatgpt.com)) |
| **Institute of Science Tokyo, Japan** | The dental curriculum was reformed partly in response to Japan’s rapidly ageing society and the increasing complexity of healthcare. | Ageing-related preparation is incorporated within an integrated medical–dental undergraduate curriculum and comprehensive patient-care training rather than a publicly named standalone geriatric course. | The curriculum promotes cooperation among multiple professions, shared medical knowledge, teamwork, and comprehensive patient care. | Specific geriatric assessment tools were not identifiable from the English-language public information reviewed. | **Embedded geriatric and interprofessional content** ([TMDU Dental School](https://www.dent.tmd.ac.jp/dentistry/en.html?utm_source=chatgpt.com)) |
| **University of California, San Francisco, USA** | The DDS curriculum includes longitudinal **Principles of Interprofessional Practice** and **Introduction to Comprehensive Care** courses; no separately named geriatric dentistry course was identifiable from the public course list reviewed. | Potentially incorporated within comprehensive-care, risk-assessment, prevention, patient-evaluation, and interprofessional-practice courses. | Interprofessional practice is introduced from the first year and continues alongside comprehensive-care education. | Course-level assessment details specific to geriatric oral health were not publicly identifiable. | **Embedded comprehensive and interprofessional content; geriatric component not publicly explicit** ([UCSF School of Dentistry](https://dentistry.ucsf.edu/programs/dds/curriculum?utm_source=chatgpt.com)) |
| **Karolinska Institutet, Sweden** | A former standalone **Geriatric Dentistry, 4.5 credits** course is listed as cancelled. Current orofacial-medicine teaching retains gerodontology and geriatric-dentistry literature, suggesting that relevant content has been incorporated into broader teaching. | Current provision appears to be embedded within orofacial medicine, adult clinical dentistry, medically compromised-patient care, and related courses rather than maintained as the earlier standalone course. | Current course documentation supports adult clinical education and interdisciplinary geriatric literature, but a defined interprofessional geriatric placement was not established from the public information reviewed. | Current relevant courses use pass/fail grading; the precise geriatric-specific assessment was not identifiable. | **Embedded geriatric content; previous standalone course discontinued** ([Karolinska Institutet Education](https://education.ki.se/course-syllabus/2TL037?utm_source=chatgpt.com)) |
| **University of Zurich, Switzerland** | The university has a dedicated **Clinic of General, Special Care and Geriatric Dentistry** providing care for older adults, adults with special needs, medically complex patients, institutionalized older adults, and care-dependent community-dwelling older adults. | The dental programme acknowledges the need to prepare students for increasingly complex medical conditions associated with demographic ageing. However, the precise undergraduate placement of geriatric teaching was not sufficiently detailed in the public curriculum information reviewed. | The clinical infrastructure includes comprehensive treatment, special-care dentistry, mobile dental services, and care for institutionalized and community-dwelling older adults. | Undergraduate geriatric-specific assessment was not identifiable from public sources. | **Specialist geriatric clinical infrastructure; undergraduate curricular placement unclear** ([ZZM UZH](https://www.zzm.uzh.ch/de/studenten/zahnmedizinstudium/curriculum/bachelor.html?utm_source=chatgpt.com)) |
| **University of Bern, Switzerland** | The School of Dental Medicine includes a **Department of Reconstructive Dentistry and Gerodontology** within an integrated five-year dental programme. | The programme describes integrated, synoptic training across dental disciplines, but the public English-language information reviewed did not specify a distinct gerodontology course, sequence, or longitudinal strand. | The programme has a strong practical orientation and integrated training, but geriatric-specific clinical or interprofessional activities were not publicly detailed. | No geriatric-specific assessment information was identified. | **Gerodontology represented institutionally; curricular detail not identifiable** ([zmk bern – Zahnmedizinische Kliniken](https://www.zmk.unibe.ch/studies/index_eng.html?utm_source=chatgpt.com)) |
| **Harvard University, USA** | The predoctoral curriculum includes the **Predoctoral Oral Health Education and Training for an Aging America** initiative, integrating geriatric oral health throughout the four-year DMD curriculum. | The programme includes classroom learning, simulation, experiential activities, clinical instruction, and enhanced exposure to older-adult care. | The initiative includes age-simulation activities and preparation for the complex medical, functional, and communication needs of older adults; it is designed as a longitudinal curricular enhancement. | The official description confirms integrated didactic and experiential learning, although detailed scoring instruments were not publicly reported on the reviewed page. | **Explicit longitudinal geriatric curriculum** ([Harvard School of Dental Medicine](https://www.hsdm.harvard.edu/news/2026/05/treat-aging-population-hsdm-enhances-geriatric-training-dental-students?utm_source=chatgpt.com)) |
| **University of Gothenburg, Sweden** | The dental programme includes a named **Gerodontology/Geriatric Dentistry** course worth 4 ECTS credits. | The course is positioned in Year 5 and addresses normal and pathological ageing, dental care of older adults, and social-odontological aspects of ageing. | The course is interdisciplinary and includes medical, nursing, social-science, ethical, functional, and healthcare-system perspectives. Teaching includes lectures, case-based group work, and practical exercises. | Assessment includes case presentation, compulsory practical group work, and an individual written examination. | **Explicit geriatric course with defined assessment** ([Göteborgs universitet](https://www.gu.se/en/study-gothenburg/geriatric-dentistry-tp0ger/syllabus/99bdb3d8-2955-11ef-a2a0-4c1db4504bb5?utm_source=chatgpt.com)) |
